# Death of Professor Agassiz

**Published:** 1874-01

**Authors:** 


But lately the telegraphic wire, and, in quick succession the press, brings us intelligence of the death of one of the foremost scientists of our age, Louis John Randolph Agassiz, who expired—says the Boston Medical and Surgical Journal—at Cambridge, Mass., after a short illness, on the evening of Sunday, December 14th, in the sixty-seventh year of his age.

"Agassiz was born in 1807, in Switzerland, in the parish of Mottier, between the lakes of Neufchatel and Morat. During his boyhood he was remarkable for scholastic learning as well as for the study of plants and animals. When only seventeen he began at Zurich the study of medicine, which he pursued at Heidelberg and Munich, where, in 1831, he obtained the degree of M.D. His life as a medical student appears to have been protracted by the interest he took in kindred branches of science, and particularly by the time given to assist Martins in the icthyological part of his work on Brazil, and later, to write his own work on the 'Natural History of the Fresh-water Fishes of Europe.' In 1832 he was aypointed Professor of Natural History at Neufchatel, where he remained fourteen years, with the exception of the time given to some journeys. It was here that he conceived and elaborated the idea of the 'glacial theory,' with which his name will be forever associated.

"He came to America in 1846, when in the prime of life, with a large stock of experience, and with his abilities fully developed, while his force and enthusiasm were unabated; the latter, indeed, only left him with life. The business on which he came being dispatched, he decided to remain, influenced chiefly by the offers of Professor Bache, of the Coast Survey, which enabled him to study the marine fauna all along the shore. The next year saw the foundation of Lawrence Scientific School, at Cambridge, where, having accepted the Professorship of Zoology and Geology, Agassiz made his home for the future. Since then his life has been one of constant study and of work at its great object, the Museum of Comparative Zoology, which, though yet in its infancy, is already a noble monument to his memory."

Quite recently one of the great illustrated weeklies has elaborately described and pictured his new Laboratory and School of Natural History, which is located upon a small island on the coast of Massachusetts. It will be fresh in the recollection of our readers. Illustrative of his all-absorbing devotion to his beloved idol, natural history, we give the following anecdote, as we heard it, not vouching for its authenticity, viz: Agassiz was lecturing upon zoology before a miscellaneous audience, in a country town of New England, when he was accosted by a *sharp* son of the soil with, "say, Professor, what a pity, that with all your vast knowledge of science, you have not mingled a little business sense. What a stupendous fortune you could make in a few years; could'nt you?" "You forget, my friend," replied Agassiz, "I am entirely *too busy to make money*, somebody else must do that."

"Professor Agassiz was celebrated for his amiability and charming personal qualities, which he possessed to a degree which is rarely joined to such indefatigable perseverance and steadfastness as his. Unsparing of himself in his work, he was an exacting leader, but a sympathetic and encourging instructor. His death leaves a place vacant in the scientific world, and in the community, which it will be difficult to fill."

